# Variable number tandem repeats of a 9-base insertion in the N-terminal domain of severe acute respiratory syndrome coronavirus 2 spike gene

**DOI:** 10.3389/fmicb.2022.1089399

**Published:** 2023-01-04

**Authors:** Tetsuya Akaishi, Kei Fujiwara, Tadashi Ishii

**Affiliations:** ^1^Department of Education and Support for Regional Medicine, Tohoku University, Sendai, Japan; ^2^COVID-19 Testing Center, Tohoku University, Sendai, Japan; ^3^Department of Gastroenterology and Metabolism, Nagoya City University, Nagoya, Japan

**Keywords:** BLAST search, insertions/deletions, GISAID, N-terminal domain, variable number tandem repeats, spike gene, severe acute respiratory syndrome coronavirus 2

## Abstract

**Introduction:**

The world is still struggling against the pandemic of coronavirus disease 2019 (COVID-19), caused by severe acute respiratory syndrome coronavirus 2 (SARS-CoV-2), in 2022. The pandemic has been facilitated by the intermittent emergence of variant strains, which has been explained and classified mainly by the patterns of point mutations of the spike (*S*) gene. However, the profiles of insertions/deletions (indels) in SARS-CoV-2 genomes during the pandemic remain largely unevaluated yet.

**Methods:**

In this study, we first screened for the genome regions of polymorphic indel sites by performing multiple sequence alignment; then, NCBI BLAST search and GISAID database search were performed to comprehensively investigate the indel profiles at the polymorphic indel hotspot and elucidate the emergence and spread of the indels in time and geographical distribution.

**Results:**

A polymorphic indel hotspot was identified in the N-terminal domain of the *S* gene at approximately 22,200 nucleotide position, corresponding to 210–215 amino acid positions of SARS-CoV-2 S protein. This polymorphic hotspot was comprised of adjacent 3-base deletion (5′-ATT-3′; Spike_N211del) and 9-base insertion (5’-AGCCAGAAG-3′; Spike_ins214EPE). By performing NCBI BLAST search and GISAID database search, we identified several types of tandem repeats of the 9-base insertion, creating an 18-base insertion (Spike_ins214EPEEPE, Spike_ins214EPDEPE). The results of the searches suggested that the two-cycle tandem repeats of the 9-base insertion were created in November 2021 in Central Europe, whereas the emergence of the original one-cycle 9-base insertion (Spike_ins214EPE) would date back to the middle of 2020 and was away from the Central Europe. The identified 18-base insertions based on 2-cycle tandem repeat of the 9-base insertion were collected between November 2021 and April 2022, suggesting that these mutations could not survive and have been already eliminated.

**Discussion:**

The GISAID database search implied that this polymorphic indel hotspot to be with one of the highest tolerability for incorporating indels in SARS-CoV-2 S gene. In summary, the present study identified a variable number of tandem repeat of 9-base insertion in the N-terminal domain of SARS-CoV-2 S gene, and the repeat could have occurred at different time from the insertion of the original 9-base insertion.

## 1. Introduction

The pandemic of coronavirus disease 2019 (COVID-19), caused by severe acute respiratory syndrome coronavirus 2 (SARS-CoV-2), is still ongoing worldwide still in end of 2022 (Alexandridi et al., 2022; Biancolella et al., 2022). The pandemic has been sustained in the last 3 years, driven by the intermittent emergence of consequential variant strains (Papanikolaou et al., 2022; Viana et al., 2022). By now, the lineages of the variant strains have been classified mainly based on the types of point mutations in the spike (*S*) gene of the virus. This is reasonable because SARS-CoV-2 Sprotein has been known to play major roles in binding to the receptor angiotensin-converting enzyme 2 (ACE2) and also as the target antigen of most neutralizing antibodies (Liu et al., 2020; Zhang et al., 2020; Min and Sun, 2021). Recently, the genomes of many SARS-related coronavirus species, including SARS-CoV-2 from humans, have reported to incorporate many mutation hotspots with relatively long and highly divergent insertions/deletions (indels; Akaishi, 2022b; Akaishi et al., 2022b), which are not common in many of other virus families (Willemsen and Zwart, 2019; Akaishi, 2022a). These indel hotspots with highly divergent RNA sequences in SARS-related coronavirus species were identified to be clustered in several specific genome positions, including the non-structural protein 2 (Nsp2) and Nsp3 of the open-reading frame 1a (*ORF1a*), N-terminal domain (NTD) of *S* gene, and *ORF8* gene (Akaishi et al., 2022a). Many of these divergent and complex indel hotspots are away from the known genomic recombination sites in the viruses (Alexandridi et al., 2022; Lytras et al., 2022). However, the genomic regions and patterns of highly polymorphic indel sites in the genomes of SARS-CoV-2 from humans have not been enough evaluated until now. Moreover, the geographical distributions and prevailing periods of each indel pattern remains largely unevaluated. Therefore, in this report, we searched for the polymorphic indel hotspots in the genomes of SARS-CoV-2 collected from humans and estimated the time period and geographical locations of the emergence of such polymorphic indels. Furthermore, we are going to report an insertion site with variable number tandem repeat of 9-base insertion, found in the NTD of the SARS-CoV-2 *S* gene.

## 2. Materials and methods

### 2.1. Initially evaluated genome sequences

In this study, a total of 20 SARS-CoV-2 genome sequences from different timings and countries were initially collected to search for the presence of polymorphic indel sites, which were randomly selected from the NCBI GenBank Database in October 2022, based on the sample collection time and geographic distribution. These sequences were first used to preliminarily search for the location of the polymorphic site across the SARS-CoV-2 genome in the last 3 years. The list of the initially collected 20 sequences is shown in Table 1 (Holland et al., 2020; Wu et al., 2020; Wilkinson et al., 2021; De Marco et al., 2022).

**Table 1:** List of the initially recruited 20 SARS-CoV-2 strains.

GenBank accession ID	Collection date	Country	Sequence names
MN908947	December 2019	China	Wuhan-Hu-1 (original)
ON507065	January 17, 2022	Italy	SARS-CoV-2/human/ITA/ID6_170122/2022
MT339039	May 17, 2020	United States	SARS-CoV-2/human/United States/AZ-ASU2922/2020
MT844030	July 20, 2020	Brazil	SARS-CoV-2/human/BRA/RJ-DCVN4/2020
OP699312	October 09, 2021	United States	SARS-CoV-2/human/United States/WA-S21827/2021
OW981938	May 07, 2022	Switzerland	hCoV-19/Switzerland/SG-ETHZ-674753/2022
OL989090	April 26, 2021	Philippines	SARS-CoV-2/human/PHL/210430–1/2021
OP355305	January 15, 2022	India	SARS-CoV-2/Homosapiens/IND/EPI_ISL_11887846/2022
OL989098	July 05, 2021	Argentina	SARS-CoV-2/human/ARG/210711–54/2021
OP024160	March 03, 2022	Japan	SARS-CoV-2/human/JPN/HiroC311c/2022
ON513706	January 17, 2022	United States	SARS-CoV-2/human/United States/TG996464/2022
ON819429	January 07, 2022	Australia	SARS-CoV-2/human/AUS/QIMR01/2022
OM945722	February 11, 2022	Turkey	SARS-CoV-2/human/TUR/ERAGEM-OM-1104/2022
ON032859	January 25, 2022	Russia	SARS-CoV-2/human/RUS/Altufjevo/2022
OM640073	January 19, 2022	Austria	SARS-CoV-2/human/AUT/SKV-316/2022
OM773467	January 19, 2022	South Africa	SARS-CoV-2/human/South Africa/NHLS-UCT-LA-Z842/2022
OP107796	July 01, 2022	Brazil	SARS-CoV-2/human/BRA/LACENAL-270228624/2022
OP279916	July 15, 2022	South Africa	SARS-CoV-2/human/ZAF/NHLS-UCT-LA-ZB06/2022
OP430898	2022	Germany	SARS-CoV-2/human/DEU/C63/2022
ON966115	March 31, 2022	Thailand	SARS-CoV-2/human/THA/BKK-ST023.8/2022

The initially recruited 20 SARS-CoV-2 sequences were randomly selected from NCBI GenBank Database, according to the sample collection time and geographical distribution. The countries of the sequences were selected to cover all of the five continents. These initially selected 20 sequences were aligned to screen for the presence of polymorphic indel sites in the genomes of SARS-CoV-2, developed during the COVID-19 pandemic since 2019.

### 2.2. Multiple sequence alignment

By using the initially collected 20 virus genome sequences, multiple sequence alignment was performed by using Molecular Evolutionary Genetics Analysis Version 11 (MEGA11) software (Tamura et al., 2021). The Multiple Sequence Comparison by Log-Expectation (MUSCLE) program was run to align the whole genome sequences. As for the alignment parameters, gap opening penalty score was set with −400 and gap extension penalty score was set with 0. The presence of polymorphic indel sites were manually searched across the whole genomes using the aligned sequences. Polymorphism of the indel site was determined if more than two patterns of indels at the indel site, including the nearby sequences of ±10 bases, were observed. Point mutation patterns in the indel sites were not considered to decide the polymorphism of the indels.

### 2.3. Basic Local Alignment Search Tool (BLAST) search

The identified sites of polymorphic RNA sequences based on sequence alignment were further evaluated by performing sequence search with NCBI basic local alignment search tool (BLAST) to know the numbers of registered sequences with 100% sequence identity with each of the identified polymorphic RNA sequence.^1^ Sequences those are 100% identical to the reference sequence were determined when they achieved 100% both for with the query cover rate and percent sequence identity. To pick up other types of overlooked polymorphic RNA sequence patterns, the identified sequences upon highly similar sequence search method (megablast) with <100% sequence identity were further checked manually and visually one by one.

Furthermore, to search for other patterns of polymorphic sequence which are not included in the initially recruited 20 sequences or are failed to be picked up by the megablast search, several patterns of hypothetical virtual sequences were prepared by gradually shortening the bases with deletion by 3 nucleotides or duplicating the bases with insertion up to 3 tandem repeats. For the collected sequences with indel polymorphisms, recombination analyses were performed using the Recombination Detection Program Version 5 (RDP5) to detect potential recombination sites across the whole virus genomes (Martin et al., 2021).

### 2.4. Global Initiative on Sharing All Influenza Data (GISAID) database search

Next, to investigate the prevalence of each observed indel type at the identified polymorphic indel site, the registered virus genome sequences worldwide were accessed *via* the Global Initiative on Sharing All Influenza Data (GISAID; Elbe and Buckland-Merrett, 2017; Shu and McCauley, 2017; Khare et al., 2021). A total of 14,066,931 genome sequences, which were registered and available up to December 1, 2022, were evaluated in the present study. The associated EPI_SET Identifier ID is specified in the subsequent data availability statement.

## 3. Results

### 3.1. Identified polymorphic site

**Figure 1:**
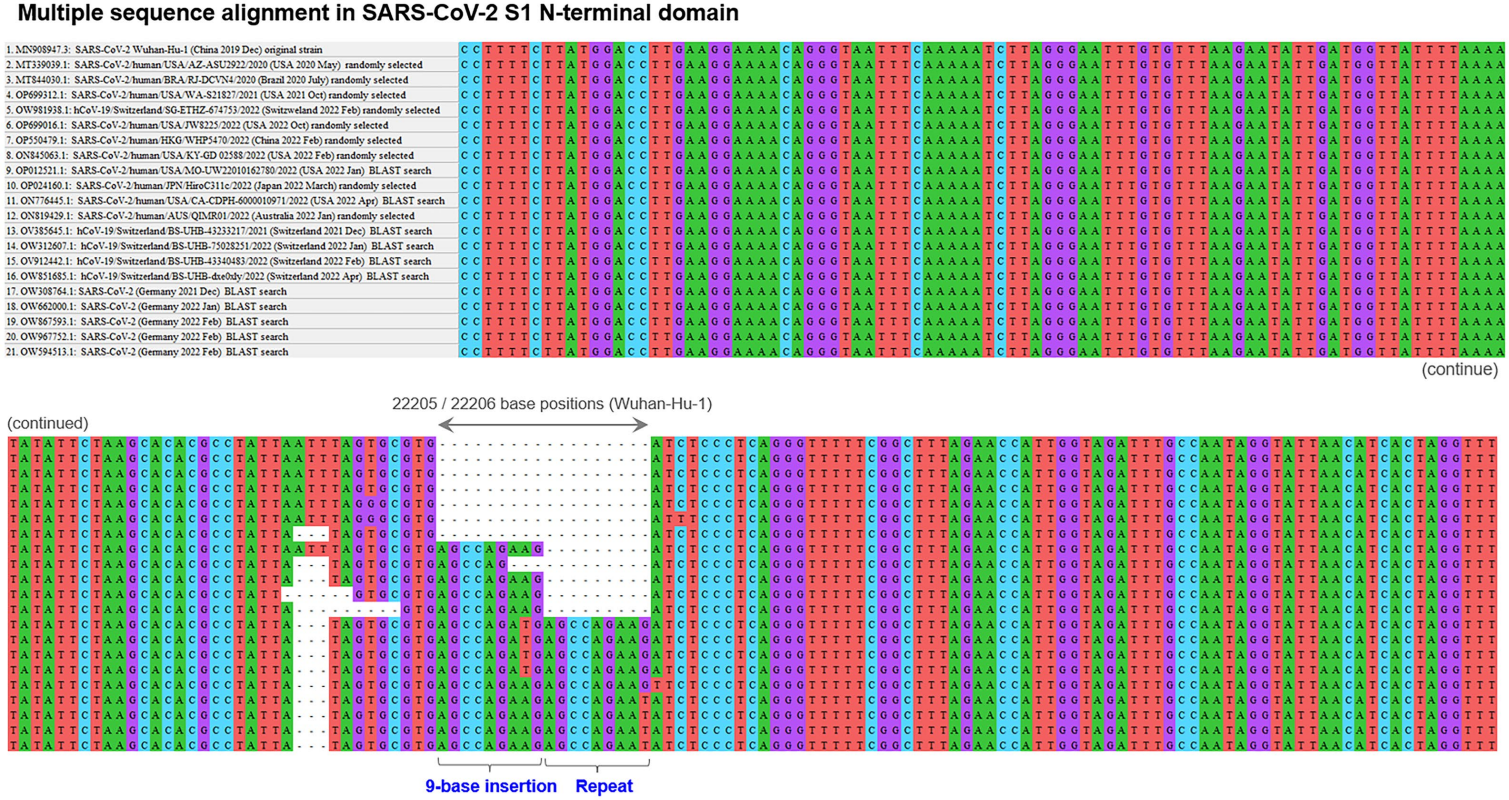
Polymorphic insertion/deletion hotspot in SARS-CoV-2 S1-NTD. The result of the multiple sequence alignment with some of the initially recruited sequences by random selection based on geographical distribution and other additional sequences identified with subsequent BLAST searches. This polymorphic indel site was comprised of two adjacent distinct indels: 3-base deletion and 9-base insertion. The combination of these 3-base deletion and 9-base insertion was confirmed in 5 of the randomly selected initial 20 sequences. Further BLAST searches with virtual RNA sequences of different indel patterns revealed the presence of SARS-CoV-2 strains with a 2-cycle tandem repeat of the 9-base insertion in the past. BLAST, basic local alignment search tool; S1-NTD, N-terminal domain of S1 gene; SARS-CoV-2, severe acute respiratory syndrome coronavirus 2.

First, the presence and location of polymorphic indel sites were screened with the initially recruited 20 virus genome sequences, which identified only one polymorphic indel site in the S1-NTD at approximately 22,190–22,210 nucleotide positions of the overall 29,903 nucleotides of SARS-CoV-2 Wuhan-Hu-1 genome. The aligned sequences around the identified polymorphic indel site with some of the randomly selected first 20 sequences are shown in Figure 1, together with the aligned sequences of some of the additional sequences detected by BLAST search. This indel site was comprised of two adjacent but distinct indels: 3-base deletion (5′-ATT-3′) and 9-base insertion (5’-AGCCAGAAG-3′). Among the initially recruited 20 sequences, both of the 3-base deletion and 9-base insertion were confirmed in the same 5 sequences, all of which were sampled and sequenced in 2022. These 5 sequences were distributed across the countries worldwide (United States, Japan, Italy, Australia, and Turkey). Sequences with these mutations accounted for 35.7% of the randomly selected sequences sampled in 2022 (*n* = 5/14 sequences). The estimated prevalence of these 3-base deletion and 9-base insertion among the viruses worldwide in 2022 was approximately 20–50%, suggesting the rapid spread of the combination of these two indels all over the globe in the early 2022. To be noted here, more than half of the randomly selected virus strains in 2022 (*n* = 9/14 sequences) still preserved the original reference RNA sequences (i.e., sequence of Wuhan-Hu-1) in this polymorphic indel hotspot site.

### 3.2. BLAST search results for the polymorphic indels

**Figure 2:**
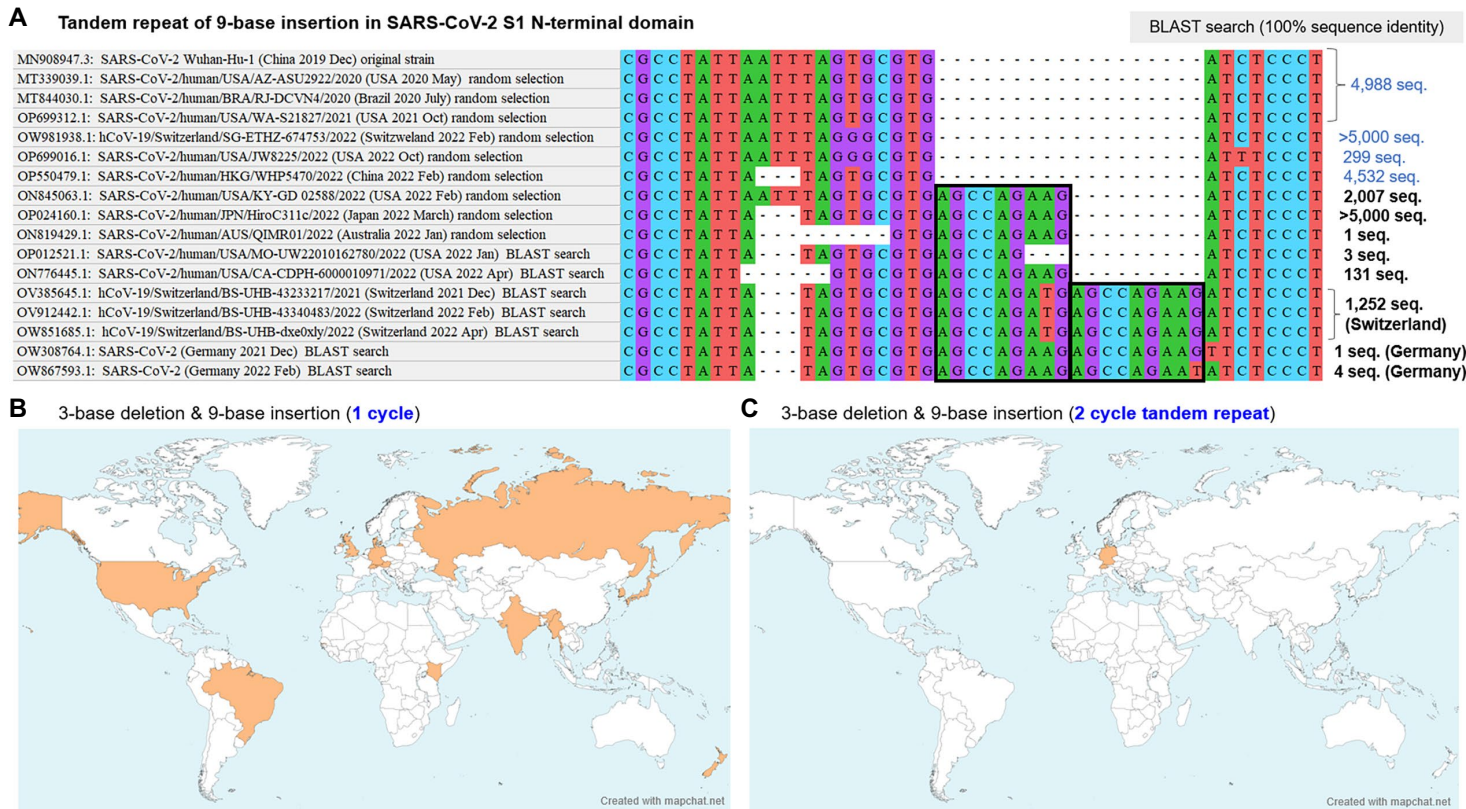
BLAST search results and geographic distribution of two-cycle tandem repeat of the 9-base insertion. **(A)** The aligned sequences at the identified polymorphic indel hotspot in SARS-CoV-2 S1-NTD are shown, together with the numbers of identified sequences with 100% sequence identity for each sequence based on the NCBI BLAST search. Both of the one-cycle and two-cycle tandem repeat of the 9-base insertion were dated from the November 2021 in Switzerland. The two-cycle tandem repeat did not spread across the globe, whereas the one-cycle 9-base insertion rapidly spread across the globe in 2022, estimated to account for 20–50% of the overall SARS-CoV-2 sampled from humans in 2022. **(B,C)** Geographic distributions of the one-cycle and two-cycles of the 9-base insertion with the nearby 3-base deletion. Although both cycles were suggested to originate in November 2021 in central Europe, the former rapidly spread across the globe, whereas the latter was limited in Switzerland. BLAST, basic local alignment search tool; S1-NTD, N-terminal domain of S1 gene; SARS-CoV-2, severe acute respiratory syndrome coronavirus 2.

Based on the finding of polymorphic indels basically comprised of 3-base deletion and 9-base insertion in the SARS-CoV-2 S1-NTD, NCBI BLAST search was performed for the identified sequences and other conceivable non-lethal virtual sequences. The search with a virtual sequence conceived from 2-cycle tandem repeat of the 9-base insertion identified a total of 1,257 registered sequences, 5 of which were from Germany and the others were from Switzerland. The presence of two different cycles of tandem repeat of the 9-base insertion exhibited the presence of variable number tandem repeats (VNTD) in RNA sequence of the SARS-CoV-2 genomes. The detailed sequences close to this polymorphic indel hotspot among the initially recruited and additionally identified sequences are shown in Figure 2A, together with the number of the identified sequences with 100% sequence identity to the entered search sequence based on the BLAST search. Recombination analysis using RDP5 was performed with these collected sequences, which did not detect any potential recombination signals across the whole virus genomes.

The identified sequences including both of the 3-base deletion (Spike_N211del) and one-cycle of 9-base insertion (Spike_ins214EPE) distributed across the countries worldwide (e.g., Germany, Switzerland, United Kingdom, Russia, United States, Kenya, Gambia, Australia, Denmark, New Zealand, India, Brazil, Myanmar, Korea, and Japan) in all five continents, as shown in Figure 2B. Based on the BLAST search, the exact time and geographical location of the emergence of Spike_ins214EPE mutation could not be determined. Meanwhile, the origin in time and location of the 18-base insertion, based on 2-cycle tandem repeat of the 9-base insertion, was more obvious because the number of the sequence was much smaller with 1,257 registered sequences. The geographical distribution of the 18-base insertion by the BLAST search result is shown in Figure 2C, most of which were collected in Switzerland since the late November 2021.

### 3.3. GISAID database search results for the polymorphic indels

To further investigate the exact time and location of the emergence of the 9-base insertion (Spike_ins214EPE) and its 2-cycle tandem repeats (Spike_ins214EPEEPE and Spike_ins214EPDEPE), we decided to perform the sequence search *via* the GISAID database. The obtained numbers of the identified sequences are listed in Table 2. More than 95% of the sequences with insertions at 214 amino acid position in the S protein were with a 9-base insertion (ins214EPE), which accounted for 13.84% of all registered sequences worldwide by November 2022. The first sample with this 9-base insertion in the GISAID database was collected in May 2020 in the United States. Regarding the 18-base insertion, we could identify two types in the GISAID database (214EPDEPE, 214EPEEPE). The first sample with ins214EPDEPE was collected in November 2021 in Switzerland, and that with ins214EPEEPE was collected in December 2021 in Brazil. The finding of the different seasons and geographical locations of the 9-base insertion and its two-cycle tandem repeat suggests that the observed set of VNTR were not created all at once, but developed gradually in different seasons at different places.

**Table 2:** Numbers of the registered genome sequences with each insertion type at Spike_214 amino acid position in GISAID database.

Insertion types	*n* (%)	Collected seasons and places
Any types of insertion at Spike_214 amino acid position	2,032,091/14,066,931 (14.45%)	March 2020 (Slovenia) – Present (worldwide)
18-base insertion (Spike_214EPDEPE)	1,259/14,066,931 (0.009%)	November 2021 (Switzerland) – April 2022 (Switzerland)
18-base insertion (Spike_214EPEEPE)	82/14,066,931 (0.0006%)	December 2021 (Brazil) – February 2022 (Brazil)
15-base insertion (Spike_214EPEEP)	0/14,066,931 (0.00%)	n.a.
12-base insertion (Spike_214EPEE)	0/14,066,931 (0.00%)	n.a.
9-base insertion (Spike_214EPE)	1,947,137/14,066,931 (13.84%)	May 2020 (United States) – Present (worldwide)
9-base insertion (Spike_214EPK)	1,133/14,066,931 (0.008%)	December 2021 (United Kingdom) – Aug 2022 (United States)
9-base insertion (Spike_214EPD)	284/14,066,931 (0.002%)	December 2021 (United Kingdom) – March 2022 (United States)
9-base insertion (Spike_214EPQ)	82/14,066,931 (0.0006%)	December 2021 (South Africa) – April 2022 (worldwide)
9-base insertion (Spike_214EPV, 214EPG)	26/14,066,931 (0.0002%)	ins214EPV: November 2021 (South Africa) – Aug 2022 (United States)
		ins214EPG: December 2021 (India) – May 2022 (United States)
9-base insertion (Spike_214EPA)	11/14,066,931 (< 0.0001%)	January 2022 (Germany) – March 2022 (Canada)
9-base insertion (Spike_214EPL)	4/14,066,931 (< 0.0001%)	ins214EPL: February 2022 (United Kingdom)
9-base insertion (Spike_214EPstop)	4/14,066,931 (<0.0001%)	January 2022 (United States) – May 2022 (United States)
9-base insertion (Spike_214EPP)	1/14,066,931 (<0.0001%)	January 2022 (United States)
9-base insertion (Spike_214EPF, EPI, EPS, EPM, EPT, EPY, EPH, EPN, EPC, EPW, EPR)	0/14,066,931 (0.00%)	n.a.
6-base insertion (Spike_214EP)	405/14,066,931 (0.003%)	Unknown
3-base insertion (Spike_214E)	361/14,066,931 (0.003%)	Unknown

The amino acids are written in one-letter code. For example, “214EPE” denotes that 9-base insertion with the resultant three amino acids insertion of “glutamic acid – proline – glutamic acid” occurred at the 214 amino acid position of the SARS-CoV-2 spike protein.

**Figure 3:**
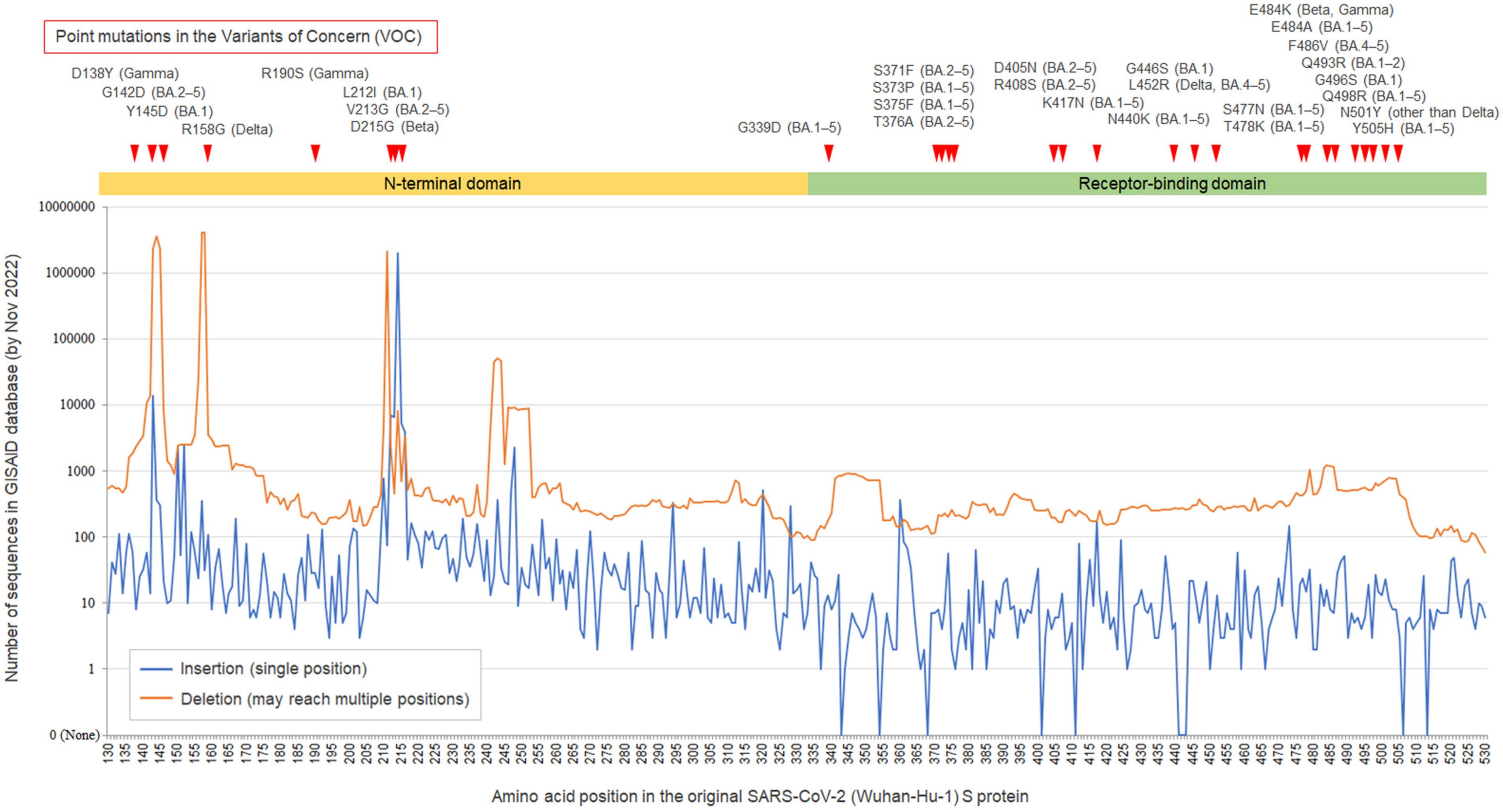
GISAID database search for the insertions/deletions in the N-terminal domain and receptor-binding domain of SARS-CoV-2 spike gene. The line graphs show the numbers of sequences sampled from humans with insertions/deletions (indels) at each amino acid position in the N-terminal domain (NTD) and receptor-binding domain (RBD) of the SARS-CoV-2 spike gene, which were registered in the GISAID database by December 01, 2022. The observed variable numbers tandem repeat of this study matches to the peak of insertion at 214 amino acid position of the S1-NTD. The asymmetrical line graphs suggest the different tolerability for incorporating indels between the SARS-CoV-2 S1-NTD and the S1-RBD, with a lower tolerability in the S1 RBD. The Y-axis scale is logarithmic.

Finally, to confirm that the finding of polymorphic insertion at the 214 amino acid position in the S protein is truly site-specific and it not common in other amino acid positions, the site-specific numbers of registered sequences in the GISAID database with insertions or deletions at each amino acid position in the S1-NTD and receptor-binding domain (RBD) of SARS-CoV-2 *S* gene are shown with line graphs in Figure 3. As can be seen, the 214 amino acid position in the S protein showed the highest peak of sequences with insertions in the evaluated 400 amino acid positions (i.e., 130–530 amino acid positions). This hotspot was also a hotspot for the point mutations in the previous variants of concern (VOCs), suggesting that this amino acid position has some potential roles for the survival of the virus and mutations at this position including indels would often function as beneficial mutations for the virus. Another notable finding of the line graphs was the asymmetrical distributions between the S1-NTD and the S1 RBD, although the frequency of point mutations in the previous VOCs was not apparently different between the two domains or even higher in the S1 RBD. This finding may suggest the different tolerability for incorporating indels between the two domains, with a lower tolerability in the S1 RBD compared to the S1-NTD.

## 4. Discussion

In this study, the presence of highly polymorphic indel hotspot in SARS-CoV-2 genomes, sampled from humans during the pandemic of COVID-19, was identified in the S1-NTD. This polymorphic indel site was comprised of the combination of adjacent 3-base deletion and nearby 9-base insertion. Furthermore, the NCBI BLAST search and GISAID database search identified several derivatives of the 9-base insertion, some of which were 18-base insertions based on two-cycle repeats of the 9-base insertion. The two-cycle tandem repeats of the 9-base insertion were suggested to have emerged in November 2021, possibly in the Central Europe including Switzerland and Germany, whereas the original one-cycle 9-base insertion could have dated back to the middle of 2020 away from the Central Europe. The two-cycle tandem repeat types have not been identified in samples collected after the April 2022, suggesting that this type of mutation could have already eliminated. Meanwhile, the one-cycle 9-base insertion type is still prevailing, known as Spike_214EPE insertion, which is one of the characteristic mutations of the Omicron variant BA.1 (Dhawan et al., 2022; Singh et al., 2022).

One of the notable findings of the present study was that it implied the possible importance of paying attention to mutations in genomic regions other than the SARS-CoV-2 S1 RBD, including S1-NTD, in monitoring and classifying the emerging consequential variant strains. Although the exact role of highly polymorphic indel site in the SARS-CoV-2 evolution and the emergence of VOCs remains undetermined, the high tolerability of S1-NTD for incorporating indels may suggest that the occurrence of polymorphic indels in this domain may be beneficial for the virus *via* some unknown mechanisms like escaping from host immunity. Another notable finding was that this study suggested the potential roles of indels and tandem repeats of inserted sequences, in addition to point mutations, in the process of SARS-CoV-2 genomic evolution. From before, VNTR has been broadly identified in the DNA sequences of the genomes in many organisms, including animals and wide variety of bacteria (Chang et al., 2007; Bilgin Sonay et al., 2015; Bakhtiari et al., 2021), but the reports of VNTR in virus genomes are currently limited (Sun et al., 1995; Avarre et al., 2011). Therefore, the process of emergence, prevalence, and potential role in virus evolution of VNTR remain largely unknown at present. The obtained results suggested that the one-cycle and two-cycle tandem repeat of the 9-base insertion emerged at different seasons in remote areas. This finding may suggest the possibility that previously inserted nucleotides in the virus genome are likely to be repeated and exhibit VNTR. While most of the extraordinarily long indels involving dozens of bases in coding regions would be deleterious and the virus with such mutations will be removed from the population, some of the tandem repeats of relatively short sequences could be non-lethal and survive in the environments, which could partially contribute to the genomic evolution of the virus. Considering from the numbers of identified sequences with the evaluated insertion types, the observed two-cycle tandem repeat of the 9-base insertion (Spike_ins214EPEEPE) and its derivative (Spike_ins214EPDEPE,) may have been non-lethal, although whether the mutations were beneficial or deleterious for the survival of the virus remains uncertain. Studies to elucidate the roles in virus evolution and exact mechanisms of tandem repeat of inserted bases are warranted.

There are several limitations for the present study. First, this study could not determine the exact process of emergence, origin in the environments, and geographical location of the original one-cycle 9-base insertion. Therefore, whether the 9-bases insertion had occurred at one time or had gradually extended by accumulations of 3-base insertion is uncertain. However, because the identified number of the 3-base insertion (Spike_ins214E) or 6-base insertion (Spike_ins214EP) was much smaller than that of the 9-base insertion (Spike_ins214EPE), it could be inferred that the insertion of the nine nucleotides had occurred at once. The environmental origin of the inserted 9-bases (AGCCAGAAG) could not be determined with BLAST search because of its short sequence length. Second, the significance of the observed VNTR for the severity of symptoms in hosts could not be estimated in this study. Determining the severity with the lineages incorporating the 2-cycle tandem repeat seems to be difficult, because the number of the registered sequences with the 18-base insertions was relatively small and the mutations have not been identified later than April 2022, as far as we could search. Lastly, although the BLAST search and GISAID database search could not identify the matched sequences to the two-cycle tandem repeat of 9-base insertion in samples collected after April 2022, this result may not necessarily mean that the tandem repeat insertion had failed to spread and had already been eliminated completely from the environments. As a possibility, the mutation may have subsequently incorporated additional mutations and the BLAST search and GISAID database search in this study could have failed to identify such possible resultant variants.

In summary, the present study identified a polymorphic indel hotspot with different tandem repeat cycles of inserted bases at the 214 amino acid position in SARS-CoV-2 S1-NTD, sampled and sequenced from humans during the COVID-19 pandemic. The obtained results implied the polymorphic patterns of indels could emerge gradually in different seasons at different geographical locations. This finding may imply that tandem repeat may be likely to occur at the indel hotspots and can repeat the previously inserted sequences. Furthermore, the tolerability for incorporating indels was suggested to be significantly different between the genomic regions and could be distinct from the distribution of tolerability for incorporating point mutations. Further studies are warranted to elucidate the potential roles of polymorphic indels and tandem repeat of insertion in the evolutionary process of viruses including SARS-CoV-2.

## Data availability statement

The original contributions presented in the study are included in the article/supplementary material, further inquiries can be directed to the corresponding author.

## Author contributions

TA and KF conceived the study, performed the analyses, and drafted the manuscript. TI supervised the study and critically reviewed and revised the manuscript. All authors contributed to the article and approved the submitted version.

## Conflict of interest

The authors declare that the research was conducted in the absence of any commercial or financial relationships that could be construed as a potential conflict of interest.

## Publisher’s note

All claims expressed in this article are solely those of the authors and do not necessarily represent those of their affiliated organizations, or those of the publisher, the editors and the reviewers. Any product that may be evaluated in this article, or claim that may be made by its manufacturer, is not guaranteed or endorsed by the publisher.

## Footnotes

^1^ https://blast.ncbi.nlm.nih.gov/Blast.cgi
